# A Sensitive Fluorescent Immunoassay for Prostate Specific Antigen Detection Based on Signal Amplify Strategy of Horseradish Peroxidase and Silicon Dioxide Nanospheres

**DOI:** 10.1155/2022/6209731

**Published:** 2022-07-20

**Authors:** Lihua Li, Wenzhi Zhang, Yan Wei, Lizhen Yu, Dexiang Feng

**Affiliations:** ^1^Department of Pharmacy, Wannan Medical College, Wuhu 241002, China; ^2^Institute of Synthesis and Application of Medical Materials, Department of Chemistry, Wannan Medical College, Wuhu 241002, China

## Abstract

A simple, sensitive, and fluorescent immunoassay for the detection of prostate-specific antigen (PSA) based on horseradish peroxidase and silicon dioxide nanospheres as a signal amplification strategy has been described. In the design, the primary antibody (Ab_1_) of PSA was first immobilized on the 96-well plates via physical adsorption between polystyrene and hydrophobic groups of the antibody molecule. The silicon dioxide nanospheres (SiO_2_ NSs), with large surface area and good biocompatibility, were loaded with horseradish peroxidase (HRP) and horseradish peroxidase-labeled secondary antibodies (HRP-Ab_2_) as signal amplification systems. In the presence of PSA, a sandwich-type immunocomplex composed of Ab_1_-Ag-HRP-Ab_2_ had been formed. Fluorescence technology was employed to obtain the response signal of the immunoassay in the *L*-tyrosine and H_2_O_2_ systems. Experiment results showed that a strong and stable fluorescent signal at 416 nm (excitation wavelength: 325 nm) was observed, and the changes in fluorescent intensity were related to the levels of PSA. Under the optimal conditions, the relative fluorescence intensity was linear with the logarithm of PSA concentration from 0.03 to 100 ng·mL^−1^, with a detection limit of 0.01 ng·mL^−1^ (at a signal/noise ratio of 3). In contrast to other fluorescent immunoassays, the sandwich-type immunoreaction based on the high binding ELISA plates has the advantages of being simple, stable, and easy to operate, high selectivity, small sample quantity, etc., which can be widely used in the selective detection of a variety of targets, from DNA to proteins and small molecules. Such fluorescent immunoassays should be feasible for the fields of molecular diagnosis and other life science fields in the future.

## 1. Introduction

Prostate cancer now ranks as one of the most prevalent health killers among males worldwide [1]. In particular, the percentages of patients in China with aggressive prostate cancer and survival rates are unpromising compared with those in western countries [2]. Prostate-specific antigen (PSA), a glycoprotein almost entirely secreted by the prostate gland, is considered to be one of the most reliable clinical biomarkers for the diagnosis, screening, and risk prediction of prostate cancer, and it was the first tumor biomarker approved by the Food and Drug Administration (FDA) [3, 4]. For healthy individuals, the serum PSA level is extremely low, about 4.0 ng·mL^−1^ and more than 10 ng·mL^−1^, making them prone to developing prostate cancer [5–7]. Therefore, the sensitive and accurate detection of serum PSA is critical for monitoring early asymptomatic prostate cancer, increasing the chances of cure and reducing its mortality.

In order to obtain ideal methods for PSA testing, scientists have proposed a series of biosensing strategies, and immunoassay was initially favored [8]. Based on the specific interaction between PSA and its antibody, immunoassays have made many achievements in PSA detecting strategies, including radioimmunoassay (RIA), enzyme-linked immunosorbent assays (ELISA), and electrophoretic immunoassay [9, 10]. The detection results of these methods are reliable. However, these methods need complex procedures and a long time. In recent years, electrochemical and optical immunoassay methods have been developed for PSA detection [11, 12]. Although low detection limits are achieved using electrochemical immunoassay, the reproducibility of electrochemical immunoassay is still a challenge. Moreover, undesired biomolecules in serum may produce interference with the detected objects. Optical immunoassays, including fluorescence (FL) [13], surface plasmon resonance (SPR) [14], chemiluminescence (CL) [15], electrochemiluminescence (ECL) [16], and surface-enhanced Raman spectroscopy (SERS) [17], overcome the disadvantages of interference. Amongst them, fluorescent immunoassay has received considerable attention due to its speed, sensitivity, and signal stability and provides a possible highly sensitive and selective determination of tumor markers at an earlier stage of disease development.

On the other hand, the growing need for early and ultrasensitive surveillance of disease-related biomarkers is driving the development of biomarker-sensitive assays through signal amplification. Fortunately, recent achievements in nanomaterials and nanotechnology have provided new avenues to develop novel signal amplification strategies for ultrasensitive immunoassays [18–20]. Because of their small size, large specific surface area, and good stability at high temperatures, nanoparticles have been widely used for signal amplifications including colloidal gold nanoparticles, magnetic nanoparticles, carbon nanospheres, quantum dots, silicon dioxide nanospheres (SiO_2_ NSs), etc. [21–25]. More importantly, the surfaces of these nanoparticles can be modified with oligonucleotides, enzymes, or antibodies to generate biological conjugates that serve as analysis cores and enhance signal generation by producing a synergistic effect to achieve higher sensitivity and lower detection limits [26, 27]. Compared with a variety of nanoparticles, SiO_2_ NSs and their nanocomposites serving as signal amplification systems have many advantages: (i) they are nontoxic and highly biocompatible with biological systems; (ii) easy to prepare and their surface can be functionally treated as needed; (iii) the performance is very stable; and (iv) lack of fluorescence quenching as noble metal, magnetic, and carbon nanomaterials [28–30].

In the present study, a sensitive and simple fluorescent immunoassay was designed for the detection for PSA. The primary antibody (Ab_1_) of PSA was first immobilized on the 96-well plates. SiO_2_ NSs was used as a carrier for the immobilization of HRP and HRP labeled secondary antibody (HRP-Ab_2_). In the presence of PSA, the immunocomplex was formed via specific recognition of antibody and antigen. The experimental results showed the introduction of SiO_2_ NSs clearly improved the immobilized amount of HRP, Ab_2_, and the fluorescent intensity of the system. In addition, the immunoassay method had a wide linear range and high sensitivity. The results were in good agreement with the reference values when the serum samples were detected by our developed method.

## 2. Materials and Methods

### 2.1. Materials and Chemicals

The 96-strips high binding ELISA plates, horseradish peroxidase-labeled monoclonal anti-PSA antibody (HRP-Ab_2_), and PSA ELISA kit were purchased from Biocell Co., Ltd., China. Horseradish peroxidase (HRP), Bovine serum albumin (BSA), 3-Aminopropyltriethoxysilane (APTES), Tetraethoxysilane (TEOS), N-hydroxysuccinimide (NHS), 1-ethyl-3-(3-dimethylaminopropyl) carbodiimide hydrochloride (EDC) were purchased from Aladdin Chemistry Co., Ltd., China. All other reagents were analytical grade and purchased from Sinopharm Chemical Reagent Co., Ltd., China. The clinical serum samples were collected from the clinical laboratory of the Yiji Shan Hospital with the consent of the patient. Twice-quartz-distilled water was used throughout the study. All experiments were performed in compliance with the relevant laws and institutional guidelines of the Ethics Committee.

### 2.2. Devices

The morphology and size of SiO_2_ NSs were measured on a transmission electron microscope (TEM, Hitachi-800, Japan). FTIR spectra of SiO_2_ NSs and functionalization were measured on an IR-21 spectrometer (Shimadzu, Japan). The UV-vis absorption spectra were measured on a U-5100 UV-vis-NIR spectrometer (Hitachi, Japan). An electrochemical impedance spectroscopy (EIS) analysis was carried out on the CHI650C three-electrode electrochemical working system. All fluorescence measurements were performed on an F-4600 fluorescence spectrophotometer (Hitachi, Japan).

### 2.3. Preparation and Functionalization of SiO_2_ NSs

The silicon dioxide nanospheres (SiO_2_ NSs) were prepared according to the modified Stöber method [31]. Briefly, 2.3 mL of TEOS was added to a mixture containing 90.0 mL ethanol, 2.7 mL ammonium hydroxide, and 5.0 mL deionized water, and reacted for 24 h under stirring. The silica colloidal dispersion was rinsed three times with ethanol and dried at 37°C to obtain monodisperse SiO_2_ NSs. 0.02 g of bare SiO_2_ NSs was then dispersed in 50 mL of ethanol and treated with 0.4 mL of APTES. After being stirred for 6 h, the suspension was centrifuged and rinsed three times with ethanol, and the amino-functionalized SiO_2_ NSs (NH_2_-SiO_2_ NSs) were obtained.

SiO_2_ NSs with functionalized carboxyl (COOH-SiO_2_ NSs) were prepared according to the reported procedure previously with a slight modification [32]. Namely, NH_2_-SiO_2_ NSs (20 mg) were suspended in 20.0 mL of N, N-Dimethylformamide (DMF) containing 0.1 mol·L^−1^ glutaric anhydride by ultrasonic stirring for 10 min and reacted 24 h under stirring. The resulting suspension was centrifuged, and the sediments were then washed three times alternately with DMF and deionized water.

### 2.4. Preparation of HRP-Ab_2_-SiO_2_ NSs Bioconjugate

**Figure 1:**
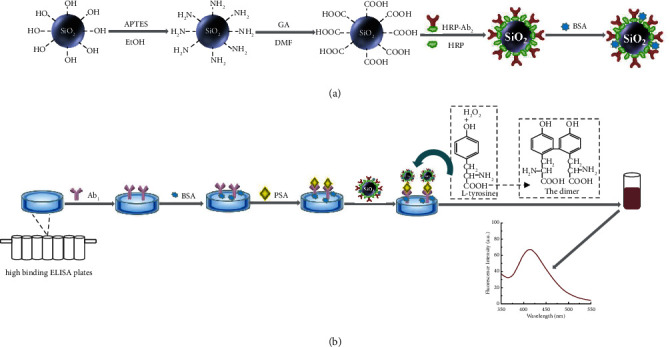
(a) Schematic illustration of the fabrication of HRP-Ab_2_-SiO_2_ NSs bioconjugate. (b) The construction process of the immunoassay for fluorescence detection of PSA.

First, the COOH-SiO_2_ NSs (20.0 mg) were suspended in 2.0 mL of PBS (pH 7.4) and ultrasonically stirred for 10 min to obtain a homogeneous dispersion. Then, 2.6 mg of EDC and 2.6 mg of NHS were added into the mixture above and activated for 30 min. Subsequently, 0.1 mL of HRP-Ab_2_ (1.0 mg·mL^−1^) and 0.3 mL of HRP (0.5 mg·mL^−1^) were added to the dispersion and stirred overnight at room temperature. The excess HRP-Ab_2_ and HRP were removed by centrifugation (10,000 rpm, 10 min). After being rinsed with PBS (pH 7.4), the resulting mixture was redispersed in 2.0 mL of PBS containing 1% BSA and stored at 4°C for further use. The schematic illustration of the preparation of the HRP-Ab_2_-SiO_2_ NSs bioconjugate is shown in Figure 1(a).

### 2.5. Detection of PSA

A sandwich-type immunoassay protocol was used for the detection of PSA as shown in Figure 1(b). First, 80.0 *μ*L of 100.0 ng·mL^−1^ PSA primary antibodies (Ab_1_) were added to the 96-well plates and placed overnight at 4°C. These wells were washed with PBS to remove unbound antibodies. Then 1% BSA was added to each well at 37°C for 45 min to block excessive binding sites. Second, 80.0 *μ*L of PSA with different concentrations was added to each well. After incubation for 1 h at 37°C, these wells were washed three times with PBS to remove the unbound antigens. Third, 80 *μ*L of the HRP-Ab_2_-SiO_2_ NSs bioconjugate was added to the wells and incubated for 1 h at 37°C. Finally, 240.0 *μ*L of Tris-HCL buffer solution (pH 7.4), 40 *μ*L of 55 *μ*M L-tyrosine, and 2.0 *μ*L of 0.003% H_2_O_2_ (5 *μ*M) were added to each well, respectively. After 30 min, the fluorescent intensity of the solution was measured.

## 3. Results and Discussion

### 3.1. Preparation and Functionalization of SiO_2_ NSs

**Figure 2:**
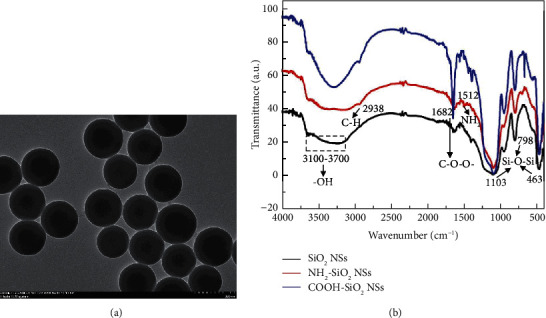
(a) TEM image of SiO_2_ NSs. (b) FTIR spectra of SiO_2_ NSs, NH_2_-SiO_2_ NSs, and COOH-SiO_2_ NSs.

In this study, TEM and FTIR were used to investigate the shape of SiO_2_ NSs obtained and their functionalization.  Figure 2(a) showed a TEM image of the SiO_2_ NSs. It could be observed that the SiO_2_ NSs had a uniform spherical shape with an average size of ∼140 nm in diameter. In the experiment, we found that the SiO_2_ NSs could be easily centrifuged, which facilitated the separation process after enzyme and antibody functionalization.

Surface modification of SiO_2_ NSs was often used in immunoassays. As a silylating reagent, APTES reacted with the silanol groups from SiO_2_ NSs to introduce amino groups to the surface of SiO_2_ NSs. The carboxylic groups were then obtained by a ring opening linker elongation reaction between the amino group and glutaric anhydride.

The FTIR spectrum of SiO_2_ NSs (Figure 2(b), black curve) displayed the presence of the asymmetrical Si-O-Si vibration (1103 cm^−1^). In addition, there were two vibration absorptions associated with Si-O-Si at 798 cm^−1^ and 463 cm^−1^, accompanied by a broad band in the range of 3100–3700 cm^−1^ of the OH stretching. In the FTIR spectrum of NH_2_-SiO_2_ NSs (Figure 2(b)), red curve), besides the absorption caused by Si-O vibrations, the weak peak at 2938 cm^−1^ was considered to be the C-H stretching vibration of the CH_2_ groups, and the 1512 cm^−1^ absorption was attributed to *δ*_NH_ of the NH_2_ groups, both of which are associated with the APTES skeleton. The signal broadening in the range of 3100–3700 cm^−1^ was attributed to an envelope of OH signal from adsorbed water, silanol, and N-H stretching vibration of the amino groups [33]. Compared with that of SiO_2_ NSs, the FTIR spectrum of the COOH-SiO_2_ NSs (Figure 2(b), blue curve) displayed a strong peak at 1682 cm^−1^, which confirmed the presence of a carboxyl group. These data indicated that the functionalization of SiO_2_ NSs with carboxyl groups was successful.

### 3.2. Characterizations of HRP-Ab_2_-SiO_2_ NSs Bioconjugate

**Figure 3:**
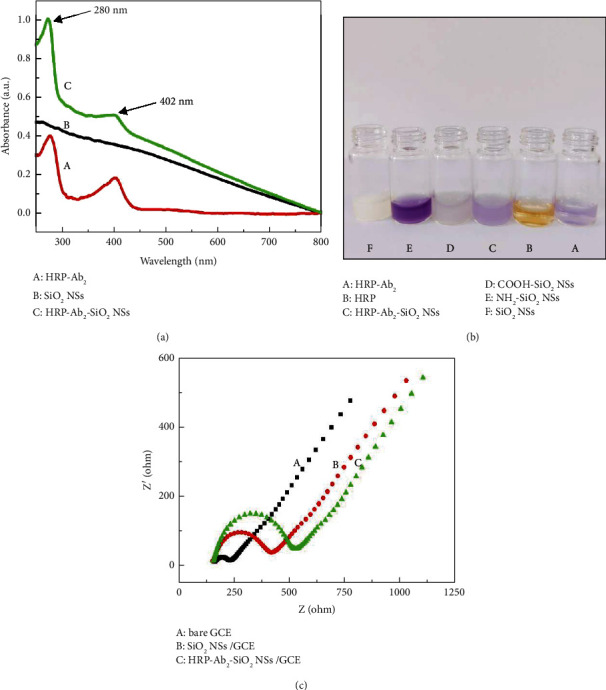
(a) UV-vis absorption of (A) HRP-Ab_2_, (B) SiO_2_ NSs, and (C) HRP-Ab_2_-SiO_2_ NSs. (b) Ninhydrin color reaction of (A) HRP-Ab_2_, (B) HRP, (C) HRP-Ab_2_-SiO_2_ NSs, (D) COOH-SiO_2_ NSs, (E) NH_2_-SiO_2_ NSs, and (F) SiO_2_ NSs. (c) EIS spectra of (A) bare GCE, (B) SiO_2_ NSs/GCE, and (C) HRP-Ab_2_-SiO_2_ NSs/GCE.

First, UV-vis spectrophotometry was used to characterize the HRP-Ab_2_-SiO_2_ NSs bioconjugates. From Figure 3(a), it could be observed that the bioconjugate appeared with two new obvious absorptive peaks at 280 nm and 402 nm, which came from Ab_2_ and HRP, indicating that HRP-Ab_2_ and HRP were successfully bonded to SiO_2_ NSs via the EDC/NHS method.

Then, the ninhydrin reaction was adopted to verify the presence of antibodies in the bioconjugate. A ninhydrin reaction is a chemical reaction in which a substance containing a free amino group, such as an amino acid, protein, or polypeptide, reacts with ninhydrin in the presence of heat and a weak acid to produce a specific-colored substance. An antibody is a protein with a unique structure that enables it to bind specifically to its corresponding antigen [34]. There are abundant amino groups and carboxyl groups in the basic structure of antibodies. As can be seen from Figure 3(b), the surface of NH_2_-SiO_2_ NSs was rich in amino groups, which reacted with ninhydrin to produce dark purple products. Compared with NH_2_-SiO_2_ NSs, HRP-Ab_2_ and HRP-Ab_2_-SiO_2_ NSs bioconjugates also contained amino groups, but fewer free amino groups, and the product color was light purple. The experimental results showed that antibodies were successfully modified on the surface of SiO_2_ NSs.

At the same time, electrochemical impedance spectroscopy (EIS) was also employed to characterize the bioconjugate. The EIS consists of a linear section representing the diffusion limiting process and a semicircular section reflecting the size of the electron transfer resistance (Ret) [35]. In comparison with the bare glassy carbon electrode (GCE, Ret about 200 Ω, Figure 3(c)) (A), the electron transfer was blocked when GCE was modified by SiO_2_ NSs due to poor conductivity, and the Ret value increased to 416 Ω (Figure 3(c)) (B). Meanwhile, it can be observed that the impedance was further improved after GCE was decorated with HRP-Ab_2_-SiO_2_ NSs bioconjugate, suggesting that the addition of antibody hindered the efficiency of the electron transfer between the electrode and the solution, which also indirectly proved that antibodies were successfully modified on the surface of SiO_2_ NSs (Figure 3(c)) (C).

### 3.3. Optimization of Immunoassay Conditions for PSA Detection

**Figure 4:**
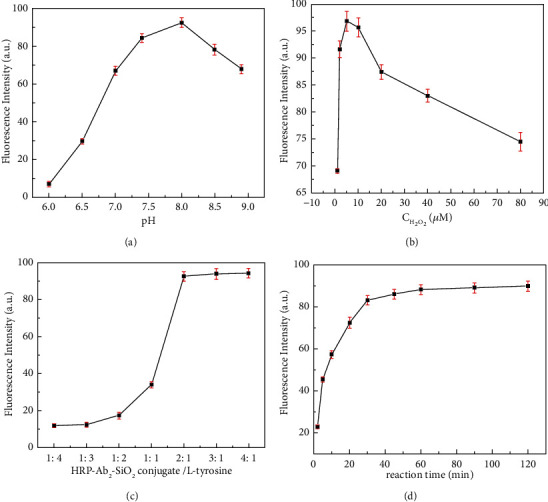
Effects of (a) pH, (b) the concentration of H_2_O_2_, (c) the ratios of HRP-Ab_2_-SiO_2_ NSs bioconjugate to L-tyrosine (v/v, L-tyrosine concentration:55 *μ*M), and (d) the reaction time on the fluorescent intensity of the immunoassay with PSA antigen concentration of 50 ng·mL^−1^ (error bars represent standard deviations from three repeated measurements).

It is well known that the results of an immunoassay are influenced by many factors, including pH and reaction time. In order to obtain excellent analytical performance, some experiment conditions were optimized. Since the pH of the solution affected HRP activity, L-tyrosine solubility, and antibody stability, we investigated the effect of pH on the immunoassay.  Figure 4(a) showed the effect of pH on the fluorescent intensity. An increase in fluorescence intensity, followed by a decline, was clearly observed as pH increased from 6.0 to 8.0. In this work, we selected a solution with a pH of 7.4 because it was close to the physiological pH value and the fluorescent intensity was relatively high at pH 7.4.

The concentration of H_2_O_2_ was a very important factor affecting the performance of the immunoassay. Low concentration resulted in an unsatisfactory performance of the immunoassay. At high concentration, horseradish peroxidase activity declined. The experiment result showed that the fluorescent intensity reached the maximum when the concentration of H_2_O_2_ was 5 *μ*M (Seen in Figure 4(b)). Therefore, 5 *μ*M of H_2_O_2_ was selected in this work.

At the same time, we investigated the different ratios of HRP-Ab_2_-SiO_2_ NSs bioconjugated to L-tyrosine because it would directly affect the signal intensity of the immunoassay. In this work, the fluorescent intensity was mainly dependent on the catalytic effect of captured HRP on L-tyrosine. If HRP content was too low, it was not enough to completely catalyze L-tyrosine, and if HRP content was too high, it would cause unnecessary waste. As can be seen from Figure 4(c), the fluorescent intensity gradually increased and then remained stable as the ratio of HRP-Ab_2_-SiO_2_ conjugate to L-tyrosine from 1 : 4 to 2 : 1. Therefore, the ratio of 2 : 1 was selected in the study, namely 80 *μ*L of the HRP-Ab_2_-SiO_2_ NSs bioconjugate and 40 *μ*L of 55 *μ*M L-tyrosine.

The effect of the reaction time on signal intensity was also investigated. As shown in Figure 4(d), the fluorescent intensity displayed its maximum when the reaction time reached 30 min and kept a stable value for 2 h at room temperature. Thus, the fluorescent measurements were carried out when the reaction time was 30 min.

### 3.4. Analytical Performance

**Figure 5:**
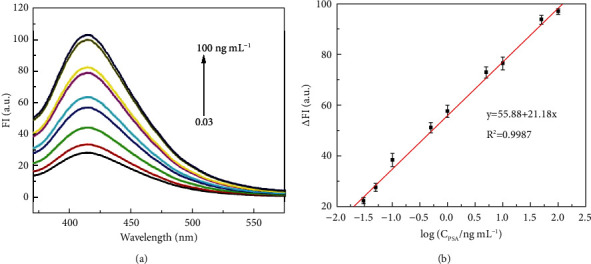
(a) Fluorescence spectra of immunoassays with a target PSA concentration of 0.03, 0.05, 0.1, 0.5, 1, 5, 10, 50, 100 ng·mL^−1^ from the bottom spectrum to the top spectrum. (b) The calibration plot of relative fluorescence intensity (ΔFI) vs. the logarithm of PSA concentration (error bars represent standard deviations from three repeated measurements).

The quantitative range of the immunoassay was assessed under optimum conditions. The results in Figure 5 showed that the fluorescent intensity increased gradually with the increase of PSA concentration, and a good calibration curve was obtained in the range of 0.03 to 100 ng·mL^−1^*y* = 55.88 + 21.18*x* was a linear regression equation, and its linear regression coefficient was 0.9987. The detection limit was estimated to be 0.01 ng·mL^−1^ (S/N = 3). The reason for the high sensitivity of this method may be attributed to the large specific surface area of SiO_2_ NSs for loading the amount of HRP and antibodies to amplify the response signal.

### 3.5. Specificity for PSA Detection

**Figure 6:**
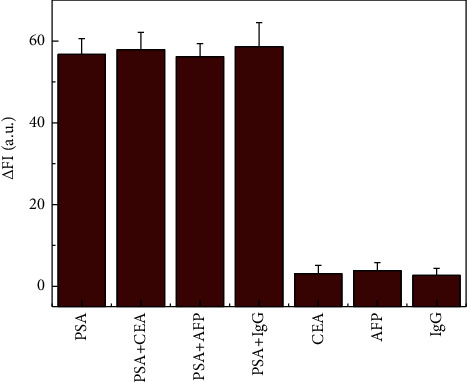
Specificity for PSA determination in the developed immunoassay. The concentration of PSA was 1.0 ng·mL^−1^, and the concentration of the other antigens was 10.0 ng·mL^−1^.

To verify the specificity of the proposed immunoassay method, similar target antigens, such as carcinoembryonic antigen (CEA), a-fetoprotein (AFP), and immunoglobulin *G* (IgG), were employed to investigate interference by monitoring the changes in the immune response signal. As shown in Figure 6, the signal produced by the non-specific antigen was negligible compared to the target antigen. The specificity of the immunoassay depends mainly upon the antibodies involved in the immune reaction. The sandwich technique with two kinds of antibodies (Ab_1_ and Ab_2_) further improved the specificity of the proposed method [36].

### 3.6. Samples Analysis

To examine the applicability of the developed immunoassay method in serum sample analysis, different quantities of known concentrations of PSA were added into the serum of healthy people for a sample recovery experiment. The results obtained were summarized in Table 1. It could be observed that the recoveries were within 96.1–105.0%, indicating that the proposed method had acceptable accuracy.

In order to further explore the feasibility of the clinical application of this method, eight human serum samples from Yiji Shan Hospital (Wuhu, China) were detected by the proposed and ELISA methods. The results were compared with the reference values obtained from the ELISA method (seen in Table 2). The relative deviations were within −5.22% to 4.16%, indicating that the results obtained by the proposed immunoassay were in agreement with those obtained by the ELISA.

## 4. Conclusions

In conclusion, a simple fluorescent immunoassay for PSA detection has been designed based on the signal amplification of HRP conjugated by SiO_2_ NSs. SiO_2_ NSs with large surface areas were loaded with more horseradish peroxidase (HRP) to speed up the catalytic process of HRP toward H_2_O_2_, which effectively amplified detection signals. The immunoassay exhibited high sensitivity and provided promising potential in clinical applications.

## Figures and Tables

**Table 1:** Recovery results of PSA in human serum samples (*n* = 9).

Serum sample	Added (ng·mL^−1^)	Found^a^ (ng·mL^−1^)	Recoveries (%)
1	0.03	0.0315	105.0
2	0.05	0.0521	104.2
3	0.1	0.1026	102.6
4	0.5	0.4805	96.1
5	1.0	0.9881	98.8
6	5.0	4.9558	99.1
7	10.0	10.214	102.1
8	50.0	48.786	97.6
9	100.0	96.366	96.4

^a^Mean value ± SD of five measurements serum sample.

**Table 2:** Comparison results of serum samples using the proposed and ELISA method.

Serum sample	Proposed method^a^ (ng·mL^−1^)	ELISA^a^ (ng·mL^−1^)	Relative deviation (%)
1	1 0.116 ± 0.012	0.113 ± 0.008	2.65
2	2 0.308 ± 0.018	0.316 ± 0.014	−2.53
3	0.653 ± 0.011	0.689 ± 0.015	−5.22
4	1.152 ± 0.009	1.138 ± 0.021	1.23
5	5.210 ± 0.025	5.002 ± 0.034	4.16
6	15.262 ± 0.138	14.806 ± 0.112	3.08
7	24.139 ± 0.165	25.418 ± 0.104	−5.03
8	43.615 ± 0.213	42.221 ± 0.226	3.30

^a^Mean value ± SD of five measurements serum sample.

## Data Availability

No additional data were used to support this study.
